# Supplementary figures and images for: Fire and Drought Affect Multiple Aspects of Diversity in a Migratory Bird Stopover Community
Source: Biology (Basel). 2025 May 24;14(6):597. doi: 10.3390/biology14060597 (PMC12189311; doi:10.3390/biology14060597)

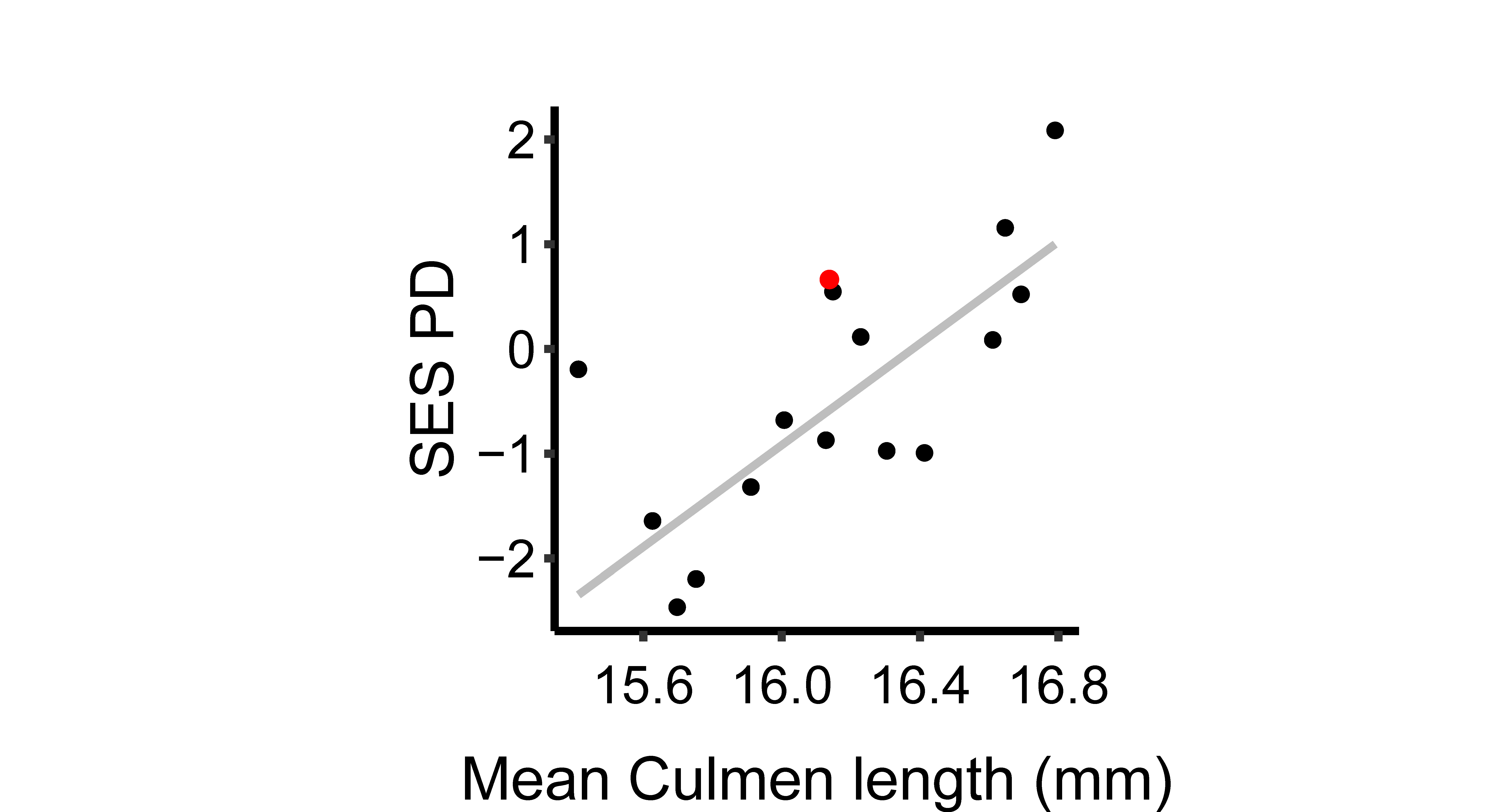

Supplement: Supplementary file 1 [file biology-14-00597-s001.zip › Figure S8 SES PD all v culmen length.tiff]

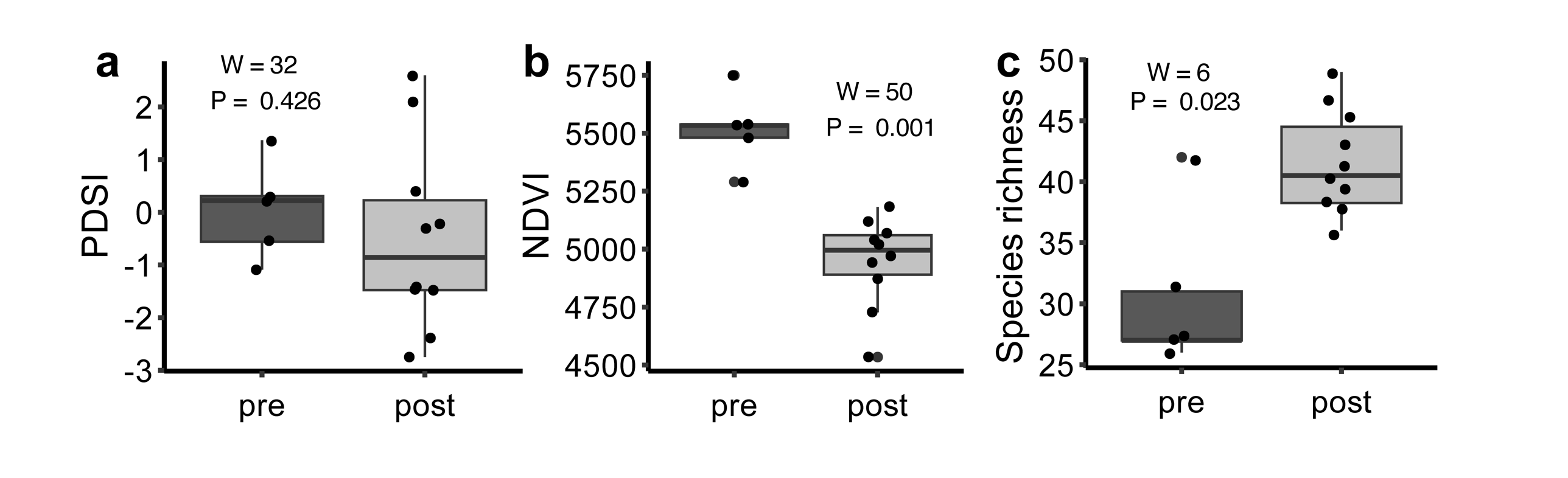

Supplement: Supplementary file 1 [file biology-14-00597-s001.zip › Figure S3 Boxplot of pre and post fire values for PDSI, NDVI, and Species Richness.tiff]

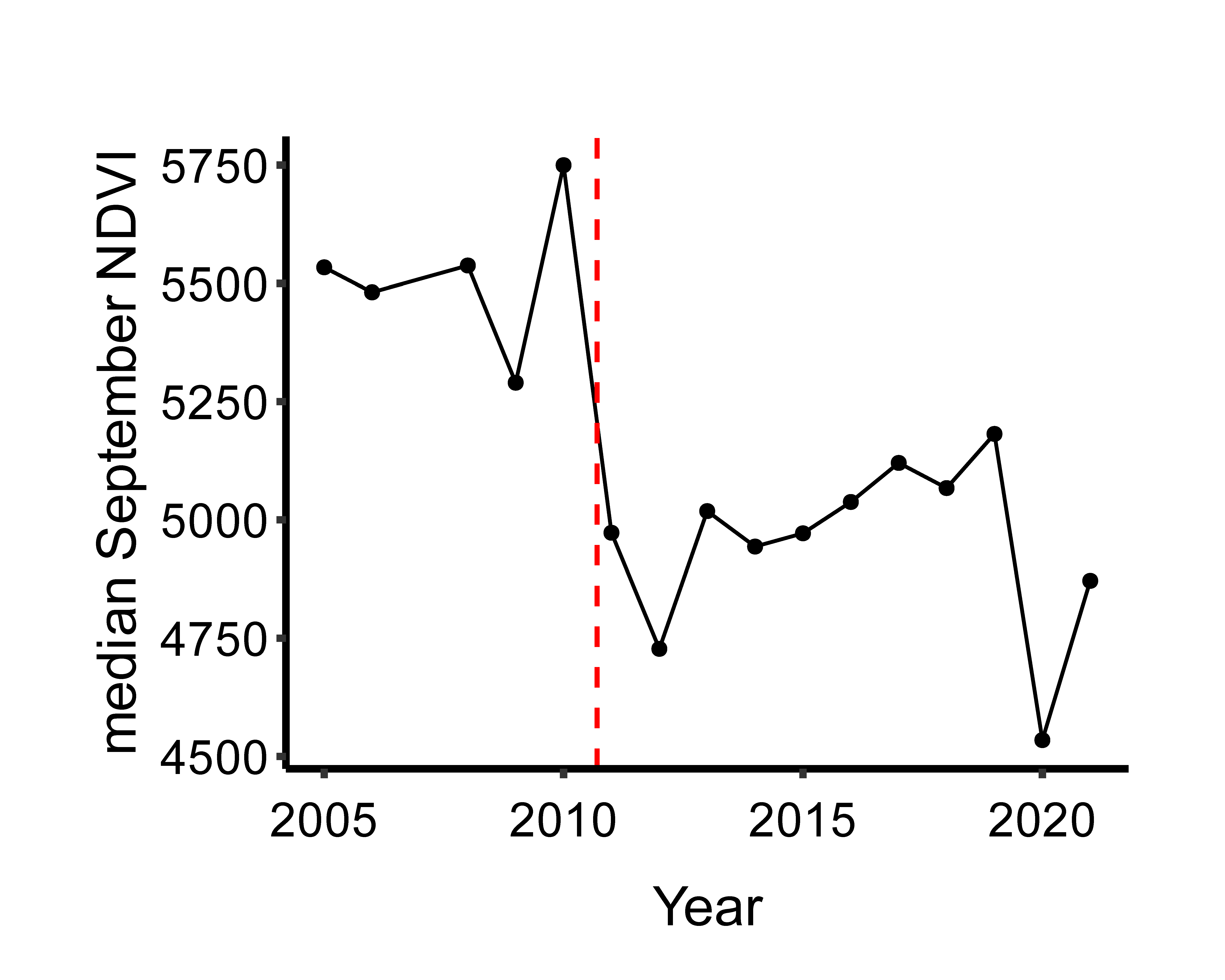

Supplement: Supplementary file 1 [file biology-14-00597-s001.zip › Figure S4 Changes in NDVI over time.tiff]

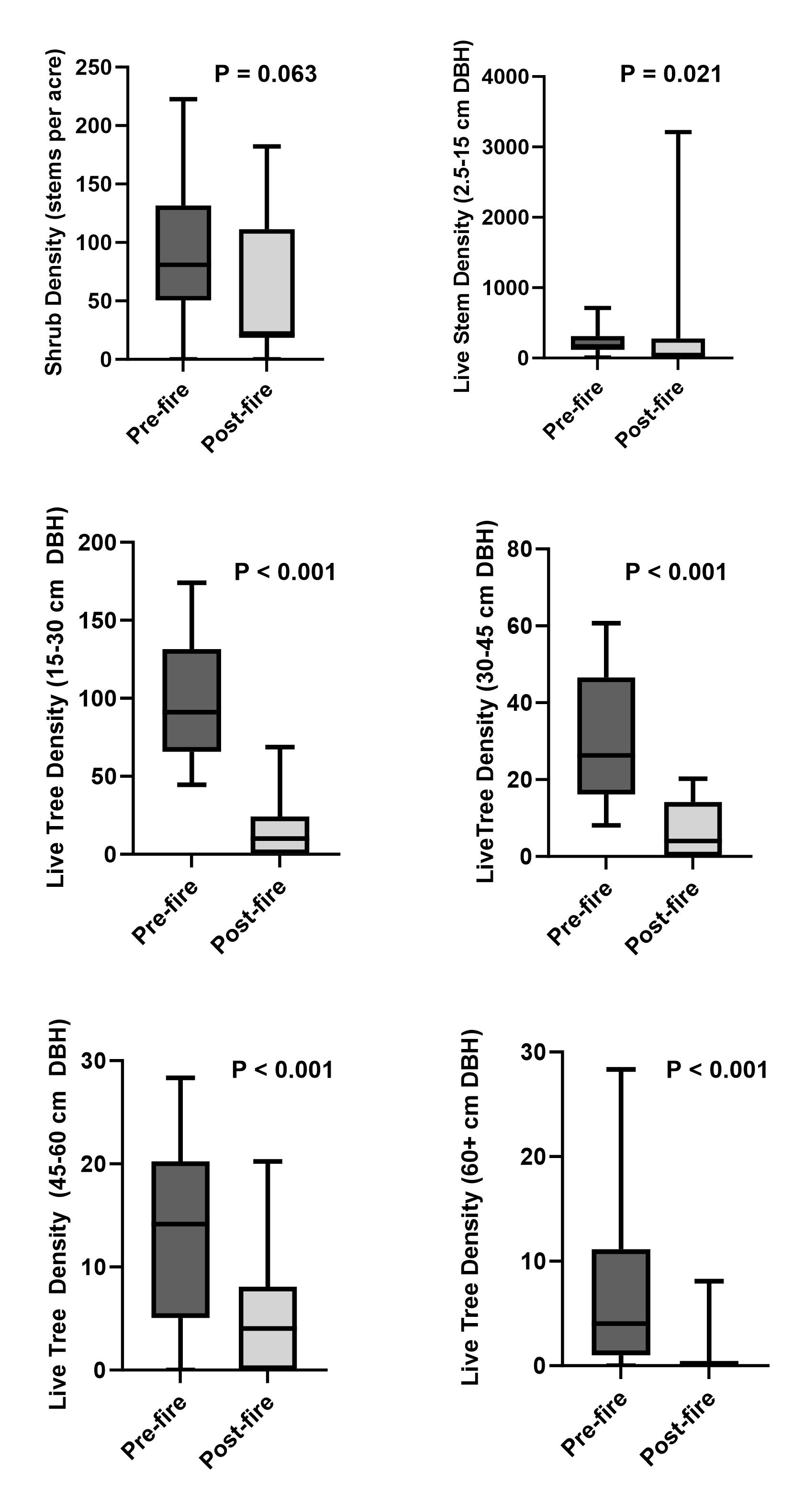

Supplement: Supplementary file 1 [file biology-14-00597-s001.zip › Figure S5 Veg box and whisker.jpg]

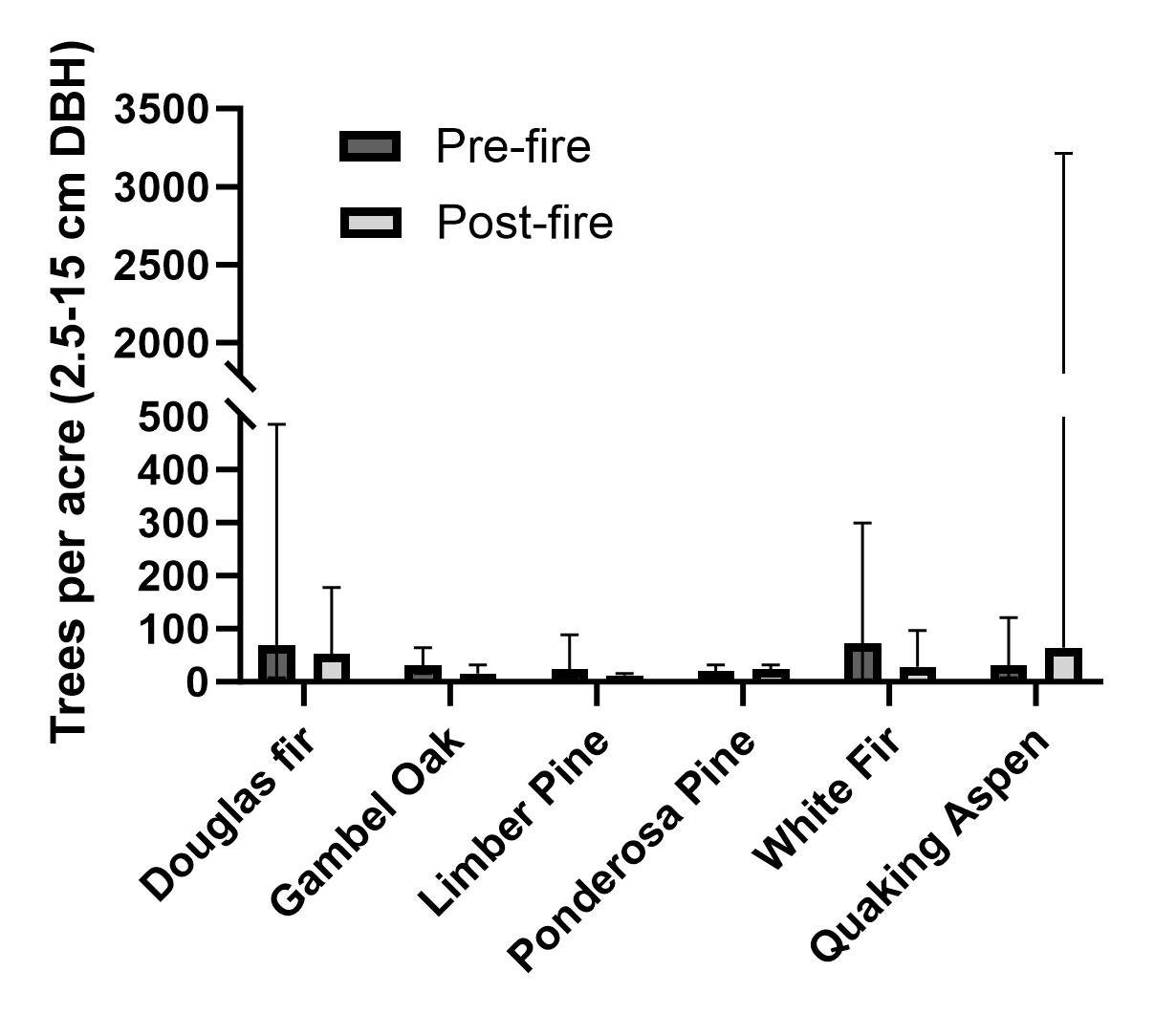

Supplement: Supplementary file 1 [file biology-14-00597-s001.zip › Figure S6 Tree Species 2.5-15 cm DBH.jpg]
